# A modest but significant effect of *CGB5* gene promoter polymorphisms in modulating the risk of recurrent miscarriage

**DOI:** 10.1016/j.fertnstert.2013.02.019

**Published:** 2013-06

**Authors:** Kristiina Rull, Ole Bjarne Christiansen, Liina Nagirnaja, Rudi Steffensen, Tõnu Margus, Maris Laan

**Affiliations:** a Human Molecular Genetics Research Group, Department of Biotechnology, Institute of Molecular and Cell Biology, University of Tartu, Tartu, Estonia; b Department of Obstetrics and Gynecology, University of Tartu, Tartu, Estonia; c The Fertility Clinic, University Hospital Copenhagen, Rigshospitalet, Copenhagen, Denmark; d Department of Obstetrics and Gynecology, Aalborg Hospital, Aalborg, Denmark; e Department of Clinical Immunology, Aalborg Hospital, Aalborg, Denmark; f Department of Bioinformatics, Institute of Molecular and Cell Biology, University of Tartu, Tartu, Estonia; g Estonian Biocenter, Tartu, Estonia

**Keywords:** Recurrent miscarriage, hCG beta coding genes, association study, *CGB5* promoter polymorphisms

## Abstract

**Objective:**

To confirm the effect of single nucleotide polymorphisms (SNPs) in *chorionic gonadotropin beta* (*CGB*) genes in modulating the susceptibility to recurrent miscarriage (RM) in Danes and in a meta-analysis across Danes and the discovery samples from Estonia and Finland.

**Design:**

Case-control association study, restriction fragment length polymorphism genotyping, resequencing.

**Setting:**

Fertility clinics at the Rigshospitalet, Copenhagen, and Aalborg Hospital, Aalborg, Denmark.

**Patient(s):**

Four hundred fifty Danish women and men from couples with RM and 119 women with children and no miscarriages in new study. A total of 634 women and men from RM couples and 314 female controls in a combined study of Estonians, Finns, and Danes.

**Intervention(s):**

None.

**Main Outcome Measure(s):**

Distribution of *CGB5* and *CGB8* allele and haplotype frequencies in patients and controls.

**Result(s):**

For the majority of studied SNPs, the allelic and haplotypic distribution differed statistically between the Danish and the previous Estonian-Finnish sample. In Danes, two *CGB5* promoter SNPs (c5-155; c5-142) exhibited a nonsignificant trend for higher allele frequency in fertile women compared with RM patients. The meta-analysis of results from three populations confirmed a modest but significant effect on carriage of c5-155C (odds ratio = 0.64; 95% confidence interval [CI] 0.44–0.94) and c5-142A (odds ratio = 0.66; 95% CI, 0.45–0.94) variants in reducing the risk of RM. None of the investigated genetic variants in the *CGB8* gene was associated with RM.

**Conclusion(s):**

Carriage of particular variants in the promoter of the *CGB5* gene seems to protect against RM. No common genetic variants in *CGB5* and *CGB8* were associated with increased RM susceptibility in the studied North European populations.

**Discuss:** You can discuss this article with its authors and with other ASRM members at **http://fertstertforum.com/rullk-cgb5-promoter-polymorphisms-recurrent-miscarriage/**

In human pregnancy, the production of hCG, a placental hormone, is indispensable. Its classical function is considered to maintain the production of steroid hormones in the corpus luteum. In addition, hCG enhances blastocyst implantation, uterine vascularization, and angiogenesis, as well as regulates maintenance of uterine quiescence and immunological adaptation during pregnancy (1–3). Low levels of hCG during the first trimester of pregnancy are related to miscarriage and extrauterine pregnancy (4–6). Abnormal circulating levels of hCG and alterations in the hormone's glycosylation patterns have been described in several pathologies (trisomies, gestational trophoblastic diseases, malignant tumors, etc.) and implied in clinical diagnostics (3, 7, 8).

A clinical condition that may develop from low hCG is recurrent miscarriage (RM), defined as three or more consecutive pregnancy losses before 22 gestational weeks (9). Apart from the known risk factors for RM (parental chromosomal anomalies, maternal thrombophilic, anatomical, endocrine, or immunological disorders), >50% of the RM cases remain classified as idiopathic (10). As the prevalence of miscarriage among the first-degree relatives of the women with RM is increased (11), a notable fraction of unexplained RM cases is expected to represent carriers of genetic risk factors involved in RM pathogenesis. Due to an irreplacable role of hCG in normal gestation, genetic variants in genes encoding hCG subunits may affect gene expression and consequently the optimal levels of hormone production as well as pregnancy success.

HCG is a heterodimeric glycoprotein consisting of two dissimilar subunits, α and β. The α-subunit gene is shared among gonadotropins (hCG, LH, FSH) and TSH, whereas the β-subunit is hormone specific. In humans, the β-subunit of hCG is coded by four duplicated and highly homologous (97%–99% DNA identity) *chorionic gonadotropin beta* (*CGB*) genes (12–15). All *CGB* genes encode identical hCG β-subunit proteins, which are critical to the level of intact circulating hCG (16). Still, the transcriptional activity among gene duplicates varies greatly, and there is also a large interindividual variation in the *hCGbeta* transcript levels (6, 17, 18). The majority, up to 82%, of the total pool of *hCGbeta* transcripts is provided by two genes, *CGB8* and *CGB5* (6, 18).

We have recently conducted a clinical resequencing study of *CGB5* and *CGB8* genes among Estonian and Finnish patients with RM and fertile controls (19). The study identified three rare variations in the protein-coding exons resulting in amino acid changes in the hCG-beta protein (Val56Leu in *CGB5*; Arg8Trp and Pro73Arg in *CGB8*), and they may therefore be potential risk factors for the occurrence of RM. The subsequent detailed functional and structural analysis of these mutations concluded that only substitutions with neutral or mild functional consequences for hCG action might be tolerated in the major hCG-beta coding genes *CGB5* and *CGB8* (20). Additionally, the resequencing described six single nucleotide polymorphisms (SNPs) in the *CGB5* and *CGB8* genes located outside the exons with significantly lower frequency among RM patients compared with the control group and thus exhibiting a protective effect towards RM (19). These polymorphisms included four linked SNPs (c5-155G→C/c5-147G→del/c5-144T→C/c5-142T→A) in the upstream of the *CGB5* gene (up to 350 bp relative to mRNA start site), which form the two main *CGB5* promoter haplotypes that are composed of the combination of either major or minor alleles of these SNPs (Fig. 1). Association with RM susceptibility was also detected for two intronic SNPs in the *CGB5* (c5+1038C→T) and *CGB8* genes (c8+1045C→T) (Fig. 1).

This study aimed [1] to confirm the effect of the *CGB5* (c5-155G→C, c5-142T→A, c5+1038C→T) and *CGB8* (c8+1045C→T) polymorphisms on the susceptibility to RM by genotyping an independent sample set from Denmark and by an extended meta-analysis across the three study populations (Estonians, Finns, Danes); [2] to resequence the promoter region of the most actively transcribed hCG-beta-coding gene *CGB8* in the Danish RM cases and controls to discover novel potential genetic risk variants to RM. The meta-analysis confirmed a modest but significant effect of the *CGB5* promoter variants c5-155C and c5-142A in reducing the risk to RM. Other investigated SNPs in the *CGB5* and *CGB8* genes exhibited no effect on RM susceptibility.

## Subjects and methods

### Study Subjects

Subjects recruited in the study were admitted to the Fertility Clinic, Rigshospitalet, Copenhagen, and the Department of Obstetrics and Gynaecology, Aalborg Hospital, Aalborg, from all over Denmark for investigation and treatment. The study sample set included 450 Caucasian patients diagnosed with RM (three or more pregnancy losses confirmed by the hospital records). The group of Danish idiopathic RM cases consisted of 199 couples and 52 single female patients. Because maternally and paternally derived gene variants contribute equally to the function of the fetal genome in placenta, the patient group included both the women and their partners who had experienced RM. In the Estonian-Finnish discovery study (19) as well as in the current Danish follow-up study, the control group was designed under the assumption that fertile women with no history of miscarriage are carrying gene variants supporting successful pregnancies. The male partners were not investigated among the control group because detailed reliable information on their reproductive history is challenging to collect. The Danish control group comprised 119 Caucasian age-matched fertile women from couples with no history of miscarriage and at least two normal pregnancies. None of the recruited female patients had uterine abnormalities found by hysteroscopy, uterine hydrosonography, or hysterosalpingography, and all RM patients and their husbands had normal karyotypes. All women were regularly menstruating with a cycle length of <35 days, and all had normal plasma thyroxin levels (detailed in [21, 22]). The study was approved by the Ethics Committees of the Capital Region, Denmark.

The subsequent meta-analysis combined the Danish data set from the current study with the discovery data of the Estonian-Finnish sample (19). The patient group of unexplained RM comprised 35 couples and 29 single female patients from Estonia and 40 couples and five single female patients from Finland. For the RM patients, the recruitment criteria in the three study centers were identical. The Estonian-Finnish control group was formed from age-matched fertile women with no history of miscarriage and consisted of 95 Estonians and 100 Finns (19). The definition of fertile female controls in the discovery study was based on at least one (Finnish) or three (Estonian) successful deliveries (the detailed description is in reference 19).

### Genotyping and Resequencing

**Figure 1:**
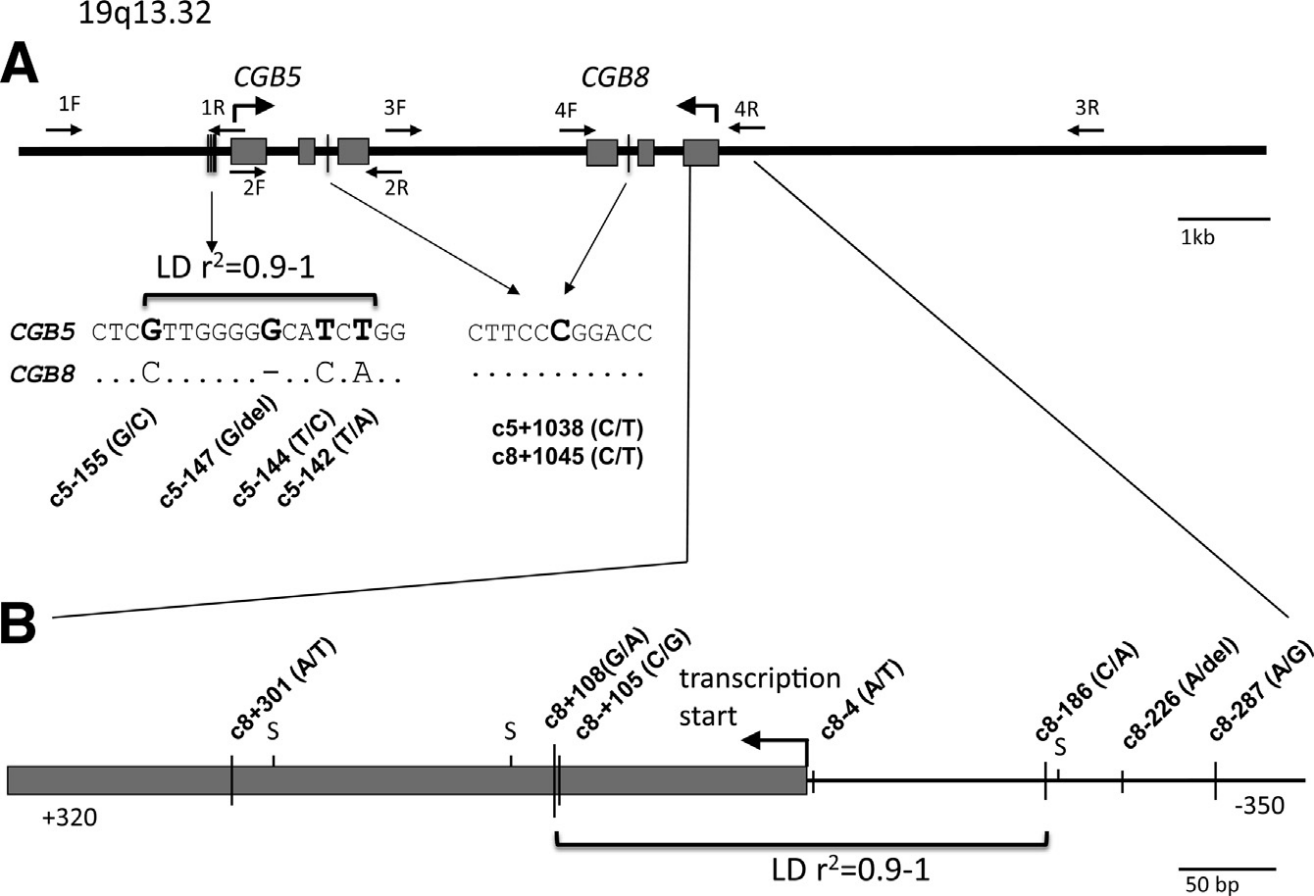
Genomic content of the studied polymorphisms in the *CGB5* and *CGB8* genes. (**A**) Design of the RFLP experiment for the genotyping of the Danish RM patients and fertile controls. The exons are depicted with *gray boxes*. A *bold arrow* shows the direction of gene transcription. The positions of the PCR primers (1F to 4F, 1R to 4R; Supplemental Table 1) for the amplification of the *CGB5* and *CGB8* genic regions are depicted with *short arrows*. The flanking regions of the genotyped SNPs (c5-155, c5-142, c5+1038, c8+1045) have been zoomed in and aligned between the two duplicate genes. *Dots* indicate identical nucleotides in the corresponding positions of *CGB5* and *CGB8*. The SNP code corresponds to the gene name (c5 = *CGB5*) and location relative to mRNA start site. The LD between the four polymorphisms in the *CGB5* promoter region is expressed using the r^2^-statistic. (**B**) The SNPs identified in Danes within the resequenced region of *CGB8* spanning the upstream region (−350 bp from mRNA start site) and the first exon (*gray box*; up to +400 bp). The proximal promoter of the *hCGbeta* coding *CGB* genes necessary for full basal expression has been demonstrated to be located between nucleotide positions −362 and +104 relative to mRNA start site (31). The direction of gene transcription is shown with a *bold arrow*. Singleton SNPs are marked with “S,” rare SNPs (MAF, <10%) with short bidirectional *vertical lines* and common SNPs (MAF ≥10%) with the long *vertical lines*. The position c8-186 is in strong LD with the SNP c8+108 in *CGB8* exon 1; r^2^ = 0.896, 0.971, and 1.0 in Danes, Estonians, and Finns, respectively. All SNPs are listed in Table 1.

DNA was extracted from peripheral blood using an in-house protocol or Puregene DNA Isolation Kit (Gentra Systems), which are both based on the salting-out method for DNA extraction. The *CGB5* (∼1.7 kb fragment) and *CGB8* (long-range polymerase chain reaction [PCR] ∼8.3 kb; nested PCR ∼2.5 kb fragment) genomic regions were amplified using previously described primers and PCR conditions (15, 19) (Fig. 1A, Supplemental Table 1).

**Supplemental Figure 1:**
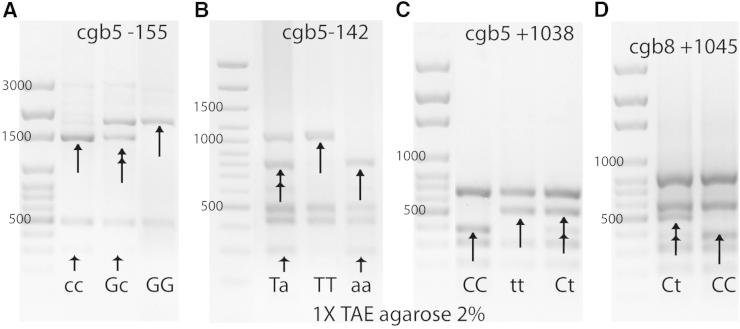
RFLP analysis to detect polymorphisms in *CGB5* and *CGB8*. (**A, B**) The PCR product of *CGB5* promoter (2243 bp) is digested with (**A**) FastDigest*StyI* (Thermo Fisher Scientific Inc./Fermentas). The substitution C/G at position −155 from the transcription start of *CGB5* gives an additional fragment of 1,449 bp; lane 1, marker 100 bp DNA Ladder (Solis Biodyne); lane 2, minor homozygote; lane 3, heterozygous individual; lane 4, major homozygote. (**B**) FastDigest*BanI* (Thermo Fisher Scientific Inc./Fermentas). The polymorphism T/A at position −142 from the transcription start of *CGB5* has an index fragment of 806 bp; lane 1, marker Gene Ruler, 100 bp DNA Ladder (Thermo Fisher Scientific Inc./Fermentas); lane 2, heterozygous individual, lanes 3 and 4, major and minor homozygotes, respectively. (**C, D**) The polymorphisms located in the same position in *CGB5* and *CGB8* (1038 bp and 1045 bp from transcription start) were addressed by digestion of PCR product of *CGB5* (1757 bp) and *CGB8* (2544 bp) with FastDigest*NciI* (Thermo Fisher Scientific Inc./Fermentas). In both graphs, lane 1 represents marker 100 bp DNA Ladder (Solis Biodyne); the index fragments of 498 bp and 308 bp allow the discrimination of the major homozygote CC (**C**, lane 2; and **D**, lane 3), heterozygous variant CT (**C**, lane 4; and **D**, lane 2), and minor homozygote TT (**C**, lane 3). Nomenclature is based on GenBank references: NM_033043.1 GI:15451747 for *CGB5*; NM_033183.2 GI:146229337 for *CGB8*; and alleles represent the nucleotides on the coding strand. The detailed restriction schema is given in Supplemental Table 2.

As the four SNPs (c5-155G→C; c5-147G→del; c5-144T→C; c5-142T→A) forming the alternative *CGB5* promoter variants are in strong linkage disequilibrium (LD) (r^2^ = 0.9–1.0; Fig. 1A), only two of them (c5-155, rs72553899; c5-142, rs72553901) were selected for genotyping as the marker SNPs for the major *CGB5* promoter haplotypes. For these two polymorphisms capturing the core *CGB5* promoter variation and for the two intronic SNPs located at the identical position within *CGB5* (c5+1038, rs4802541) and *CGB8* (c8+1045, rs4802541), the genotypes were assessed by restriction fragment length polymorphism (RFLP) analysis. Detailed information and restriction analysis scheme are shown in Supplemental Figure 1 and Supplemental Table 2.

The 5′-upstream gene regulatory region of *CGB8* was subjected to full resequencing, covering from 350 bp upstream relative to the mRNA start site to the end of exon 1 (at +400 bp). Primer design for the additional PCR amplification and sequencing primers was implemented using the Primer3 software (http://frodo.wi.mit.edu/cgi-bin/primer3/primer3_www.cgi). Sequences were resolved using ABI 3730 XL DNA Analyzer (Applied Biosystems) and analyzed by the Phred, Phrap and Consed package (23), which facilitates base calling from sequencing trace files, sequence quality assessment, and assembly. Polymorphisms were identified using the PolyPhred program (ver. 6.02.) (24) and confirmed by manual checking. A genetic variant was called only if it was observed in both forward and reverse orientations. The nomenclature of the polymorphisms was based on the following GenBank reference sequences: NM_033043.1 GI:15451747 for *CGB5*; NM_033183.2, GI:146229337 for *CGB8*.

### Data Analysis

Allele frequencies were estimated, and conformance to Hardy-Weinberg equilibrium (HWE) in the full sample as well as in patient and control subgroups was calculated by Fisher's exact test implemented in the GenePOP software package (http://genepop.curtin.edu.au/index.html) (25). The statistical tests for population differentiation comparing allele and genotype frequencies of all studied SNPs among the three populations (Danish, Estonian, Finnish) were performed using GenePOP (25).

Association with the diagnosis of RM as a binary trait was assessed by the Cochran-Armitage test for trend. Association tests and calculation of LD between SNP pairs (*r*^2^) were performed with the PLINK software, version 1.04 (http://pngu.mgh.harvard.edu/∼purcell/plink/). The LD r^2^-statistic represents the square of the correlation coefficient between the alleles at addressed loci.

For a meta-analysis including data from three recruitment centers, the inverse-variance method was implemented under a fixed-effects model using R, version 2.7.2 (R Development Core Team, http://www.r-project.org/). Odds ratios (OR) with 95% confidence intervals (CI) were calculated to show the strength and direction of the association. *P*<.05 was considered statistically significant.

Haplotypes within the resequenced region of *CGB8* (−350 bp to +400 bp relative to mRNA start) were determined based on all but singleton SNPs. Singleton polymorphisms carried in heterozygous status by one single individual were excluded from haplotype calculations as their location on either of the chromosomes cannot be reliably phased. Haplotypes were inferred from unphased genotype data using the Bayesian statistical method in the program PHASE 2.1.1 (http://www.stat.washington.edu/stephens/), applying the model allowing recombination (26). The running parameters were number of iterations = 1,000, thinning interval = 1, and burn-in = 100; the −×10 parameter was used for increasing the number of iterations of the final run of the algorithm. The relationship between inferred haplotypes was analyzed with NETWORK 4.6.1.0. software (http://www.fluxus-technology.com) using the Median-Joining network algorithm (27). Haplotype networks for *CGB8* were calculated using SNPs covering the promoter region up to the end of the first exon.

## Results

### Frequencies of *CGB5* and *CGB8* SNPs and Haplotypes Vary among North Europeans

The *CGB5* SNPs subjected to genotyping by RFLP (promoter: c5-155, c5-142; intron II: c5+1038; Fig. 1A) exhibited significantly (Fisher's exact test, *P*≤.002) lower allele frequency in Danes (n = 569; minor allele frequency [MAF], 5.94%, 5.94%, and 7.45%, respectively), compared with the published Estonian-Finnish sample (9.92%, 10.58%, and 11.38%, respectively; Table 1) (19). Within the resequenced region of *CGB8*, the allele frequencies of common SNPs (MAF >1%, c8-287, c8-186, c8+108; Fig. 1B) also differed significantly among the study samples (*P*<.05; Table 1). The genotyped SNP in *CGB8* intron II (c8+1045) was rare among Estonians-Finns (MAF 1.09%) and Danes (MAF 0.52%).

**Figure 2:**
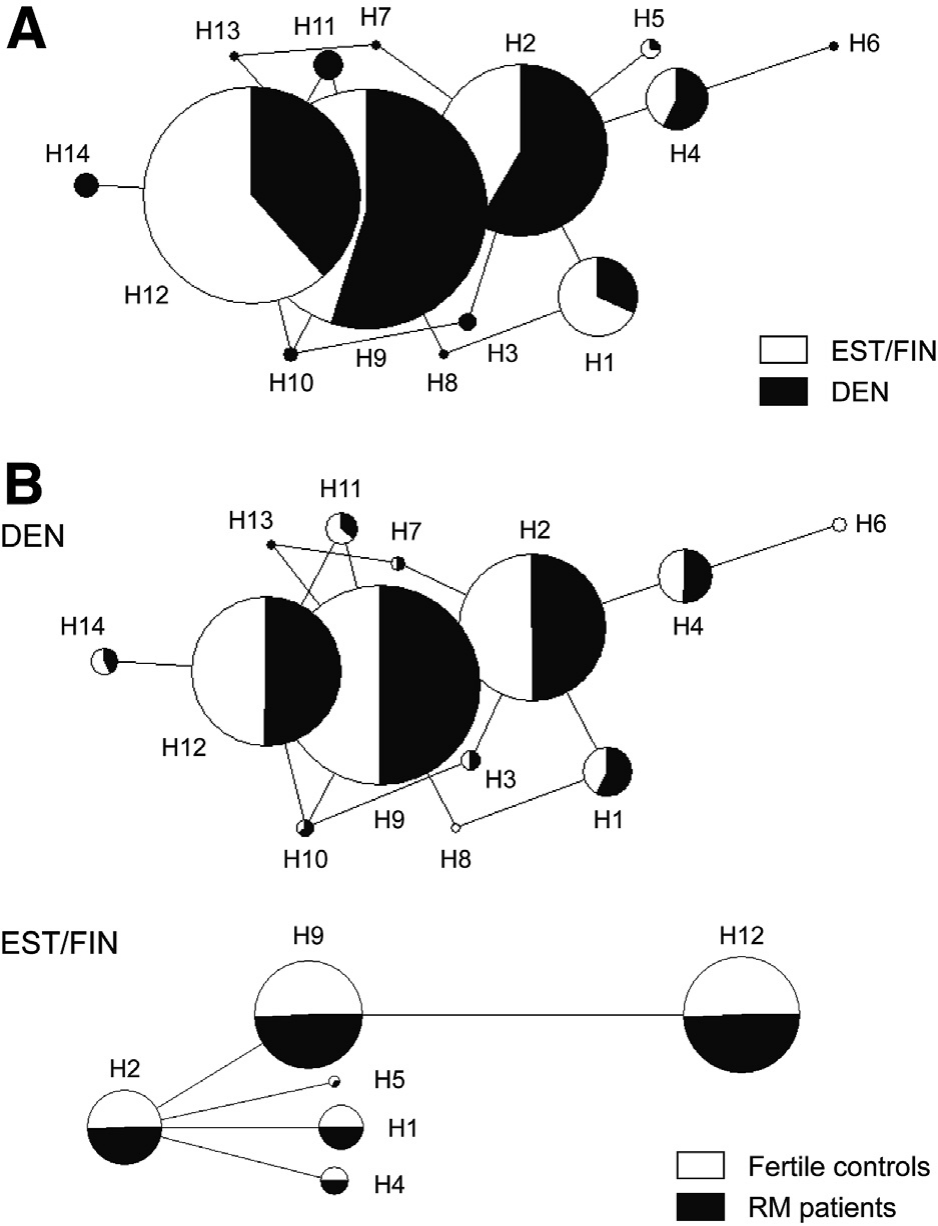
Networks of predicted haplotypes of the resequenced region of *CGB8* spanning the upstream region (−350 bp from mRNA start site) and the first exon (up to +400 bp). The size of each node is proportional to the haplotype frequency in the total analyzed data set and the length of connecting lines is proportional to the number of mutational steps between haplotypes. The nomenclature and detailed composition of haplotypes are shown in Supplemental Table 3. (A) Comparison of the haplotype distribution between the Danes (DEN; *black;* n = 569) and Estonians-Finns (EST/FIN; *white;* n = 379; [19]). The haplotypes were inferred from seven polymorphisms present more than once among the genotyped Danish-Estonian-Finnish individuals. (B) Comparison of the haplotype distribution between the recurrent miscarriage (RM) cases and fertile controls within the Danish (DEN) and the Estonian-Finnish (EST/FIN) study samples. The haplotypes in the Danes were formed from six and in the Estonian-Finnish sample from five polymorphisms, as some SNPs were population-specific or occurred as singletons in either of the analyzed study population.

Among North Europeans, the resequenced *CGB8* gene regulatory region is represented by three core haplotypes—H2, H8, and H11—determined by the allelic combinations of the two unlinked (LD r^2^ = 0.16–0.23) common polymorphisms, c8-287 and c8-186 (Fig. 1B, Fig. 2A). In total, approximately 91% of individuals in the Danish and Estonian-Finnish study samples carried the H2, H8, or H11 core haplotypes, although their distribution was statistically different among populations (*P*≤.002; Supplemental Table 3). Notably, the position c8-186 is in strong LD (r^2^>0.8) with the SNP c8+108 located in 5′UTR of *CGB8* exon 1 (Fig. 1B). It is also noteworthy that haplotype c8-287C/c8-186T combining the minor alleles of these SNPs was missing among the genotyped individuals (n = 948), although the expected carrier frequency estimated from the observed allele frequencies is ∼9%.

### Susceptibility to RM Is Modulated by *CGB5* Promoter Polymorphisms

In the Danish sample set, both genotyped SNPs in the *CGB5* promoter region (c5-155; c5-142) exhibited a higher minor allele frequency in Danish fertile women (n = 119; MAF 7.14%) compared with RM patients (n = 450; 5.62%). However, the difference was not statistically significant (*P*=.367).

To increase statistical power, the genetic data of the Danish, Estonian, and Finnish recruitment centers were combined in a meta-analysis across the three study samples (total number of 948 individuals; 634 RM patients and 314 fertile female controls; Table 2). The carrier status of the minor alleles of the *CGB5* promoter SNPs exhibited a modest but significant protective effect against RM occurrence (*P*=.021; c5-155: OR = 0.64; 95% CI, 0.44–0.94; and c5-142: OR = 0.66; 95% CI, 0.45–0.94; Table 2). This result enhanced and confirmed the outcome of the original report (19). The meta-analysis including only Danish, Estonian, and Finnish female RM patients (n = 349) compared with fertile female controls (n = 314) showed the same direction and magnitude of the effect as the analysis in the full sample, but it did not reach statistical significance owing to the smaller sample size (c5-155: *P*=.116; OR = 0.71; 95% CI, 0.46–1.08; c5-142: *P*=.089; OR = 0.68; 95% CI, 0.44–1.06). Overall, both male and female partners of RM couples had a lower prevalence of the minor alleles of the studied *CGB5* promoter SNPs (c5-155 and c5-142) compared with fertile controls (Supplemental Table 4). The allele frequencies of the genotyped intronic polymorphisms (*CGB5*: c5+1038; *CGB8*: c8+1045) did not differ between the Danish RM cases and fertile controls (MAF, 7.14% vs. 7.42%, *P*=.52; 0.55% vs. 0.43%, *P*=.83, respectively; Table 2).

### Genetic Variation in *CGB8* Promoter Does Not Affect RM Risk

The allelic distribution of SNPs (excluding singletons) in the resequenced *CGB8* gene regulatory region (from −350 bp to +400 bp from mRNA start) did not differ between the Danish RM patients and fertile controls (Supplemental Table 5), confirming the discovery analysis in the Estonian-Finnish sample (19). Concordantly, no statistical difference was detected in the *CGB8* haplotype distribution between RM patients and fertile controls either (Fig. 2B; Supplemental Table 3). We conclude that common genetic variants in the proximal regulatory region of *CGB8* have no substantial effect on the susceptibility to RM.

## Discussion

Previously, we showed a significant association between six SNPs located in the promoter region or introns of the *CGB5* and *CGB8* genes and reduced susceptibility to unexplained RM among Estonians and Finns (19). The present study set out to confirm this finding in another European population (Danes) and in a meta-analysis across the three study populations. The two discovery samples, representing neighboring populations of Estonians and Finns, had exhibited similar allelic distributions of SNPs in the *CGB5* and *CGB8* genes, whereas the allele frequencies of the Danes appeared to be statistically different from the Estonian-Finnish sample. A recent large-scale study showed that the geography of European populations is also reflected in its genetic structure, where Scandinavians cluster together with western Europeans and the Estonian population is genetically closest to Finns (28). Thus, meta-analysis rather than pooling the samples across studies is a preferred approach for increasing study power.

As a major outcome, this study confirmed the effect of the *CGB5* promoter variants on modulating the susceptibility to RM. The carrier status of the minor alleles of the two SNPs (c5-155, c5-142) investigated in the present study as the genetic markers for the *CGB5* promoter haplotypes significantly reduced the risk of RM (meta-analysis, *P*=.021, OR = 0.64 [0.44–0.94]). This RM-protective *CGB5* promoter haplotype consists of the minor alleles of four SNPs (c5-155G→C; c5-147G→del; c5-144T→C; c5-142T→A) and is completely identical to the homologous region in the *CGB8* gene, exhibiting no genetic variation in these positions (Fig. 1).

All humans have the *CGB8* promoter haplotype c8-155C/-c8147del/c8-144C/c8-142A, which seems to provide the most optimally functioning promoter because *CGB8* is responsible for up to 40% of hCG production in pregnancy (6). Most probably, originally humans had the *CGB5* gene with a slightly less efficient main promoter variant c5-155G/c5-147G/c5-144T/c6-142T (Fig. 1A). The detected *CGB5* RM-protective haplotype c5-155C/c5-147del/c5-144C/c5-142A originates from the *CGB8* gene via a meiotic gene conversion event between the two promoter regions (15). We speculate that in some pregnancies, where the trophoblast growth is impaired (due to genetic, trombophilic, immunological, or other reasons), the placenta with the most efficient *CGB5* promoter haplotype (originating from and identical to *CGB8*) may have a better capacity for extra hCG production that may eventually rescue the threatened fetuses. Subsequently, this *CGB5* promoter haplotype is expected to become increasingly prevalent among humans and to exhibit a higher prevalence in couples with normal fertility than in those with RM. This is in agreement with the results of this study. We also suggest that the current *CGB8* gene with the c8-155C/c8-147del/c8-144C/c8-142A promoter haplotype has already reached maximum efficiency. Therefore the detected common variations in this gene have neither evolutionary advantage nor effect on pregnancy success, and balancing selection is expected to rapidly eliminate new, less fit variants (19).

In conclusion, despite the essential role of hCG in human pregnancy, no common SNP or haplotype variants in the main *hCGbeta* coding genes (*CGB5, CGB8*) were associated with increased risk of RM among the analyzed North European samples. Instead, the evolution in human lineage seems to have favored the spread of *CGB* genetic variants (e.g., by gene conversion), which support a more efficient gene expression and may reduce the risk of pregnancy loss even in critical situations. Recent studies have suggested that apart from SNPs, the expression of *CGB* genes might be modified by epigenetic mechanisms (29, 30). A pilot study reported polymorphic DNA methylation in the *CGB5* promoter region exclusively in placentas from RM cases leading to expressional silencing of the paternal alleles (29). Future larger studies have to target epigenetic modifications and also other non-SNP variations (e.g., copy number variations, gene deletions/duplications) in the *CGB* genes, which may have clinical importance in modulating susceptibility to pregnancy loss.

## Figures and Tables

**Supplemental Table 1:** Primers for PCR amplification and resequencing in *CGB5* and *CGB8* genes and 5′ upstream regions.

Primer	Sequence	Product size	Fig.1 label	Original name of the primer^a^
Primers for PCR				
*CGB5* promoter				
CGB5pr_F	5′-TTTAGTAGAGACAGGGATTCACCA-3′	2243 bp	1F	
CGB5pr_R	5′-AGACCACGGTGAAGTGATCTCAG-3′		1R	
*CGB5* gene				
CGB5_F	5′-CAGGAAAGCCTCAAGTAGAGGAG-3′	1757 bp	2F	CGB5_3F
CGB5_R	5′-CGCTCGACGATGTTTTCTATTTT-3′		2R	CGB5_2R
*CGB8* long-range PCR				
CGB8_F	5′-CACGCCTGTAATTGTCGGAGGCTGT-3′	8384 bp	3F	CGB5/7_8kb_F3
CGB8_R	5′-GAAAAGAGAGTGAAGATGGGGGACGAC-3′		3R	CGB5/7_8kb_R3
*CGB8* nested PCR				
CGB8n-F	5′-CCCGGATAACTTTTCGTATTTTTA-3′	2544 bp	4F	CGB2_2R
CGB8n_R	5′-TCCTCAGATCAACTCTCATGGAT-3′		4R	CGB5/7_3nestR
Primers for resequencing				
*CGB8* promoter and 5′UTR				
cgb8prom_seqF	5′-CCCTGCAGTCTTACCTGGAA-3′			
cgb8prom_seqR	5′-TGCTGTGCCAACCTATACCC-3′			
cgb8_1F	5′-GGCCTTTGAGGAAGAGGAGT-3′			
cgb8_1R	5′-GCCTCAGGTGGTGTGCAA-3′			

a Reference 15.

**Supplemental Table 2:** List of addressed single nucleotides with applied restriction enzymes and fragment length.

SNP^a^	rs No.^b^	Allele^c^ major/minor variant	Restriction enzyme	Fragments present in all variants; lenght in base pairs	Specific fragments according to addressed nucleotide; length in base pairs		
					Major homozygous	Minor homozygous	Heterozygous
*CGB5* promoter (PCR product 2245 bp)							
c5-155	rs72553898 ss10510699321	G/C	FastDigest*StyI* (*Eco130I*)	486	1,759	310, 1,449	310, 1,449, 1,759
c5-142	rs72553901 ss105106996	T/A	FastDigest*BanI* (*BshNI*)	63, 156, 425, 492	1,109	303, 806	303, 806, 1,109
*CGB5* gene (PCR product 1757 bp)							
c5+1038	rs4802541 ss105107021	C/T	FastDigest*NciI* (*BcnI*)	7, 28, 79, 204, 305, 636	120, 378	498	498, 120, 378
*CGB8* gene (nested PCR product 2544 bp)							
c8+1045	rs4802541 ss105107049	C/T	FastDigest*NciI* (*BcnI*)	2, 7, 12, 28, 79, 204, 306, 583, 825	120, 378	498	120, 378, 498

a SNP code includes gene name (e.g., c5 = *CGB5*) and location relative to mRNA start site; GenBank references: NM_033043.1 GI:15451747 for *CGB5*, NM_033183.2 GI:146229337 for *CGB8*.

b rs number according to NCBI SNP database.

c Alleles on the coding strand. All restriction enzymes were provided by Thermo Fisher Scientific Inc./Fermentas.

**Supplemental Table 3:** The distribution of haplotypes covering the promoter and 5′ untranslated region of *CGB8* among the patients with RM and fertile controls in Danish (n = 569) and Estonian-Finnish sample sets (n = 379).

Haplotype	Position relative to transcription start site							Estonians/Finns			Danes			*P* value^a^
	−287	−226	−186	−4	+105	+108	+301	RM cases	Fertile controls	All	RM cases	Fertile controls	All	
1	c	A	G	T	G	C	A	4.25	7.33	5.85	3.14	2.52	2.95	.00569
2∗	c	A	G	T	G	C	T	16.71	16.75	16.74	23.62	23.53	23.59	.00091
3	c	A	G	T	G	t	T	0	0	0	0.37	0.42	0.38	.09237
4	c	A	G	T	c	C	T	2.27	2.36	2.31	3.14	3.36	3.20	.29044
5	c	A	G	a	G	C	T	0.85	0	0.41	0	0	0	.07409
6	c	del	G	T	G	C	T	0	0	0	0.18	0.42	0.26	.16953
7	T	A	G	T	G	C	A	0	0	0	0	0.42	0.13	.33153
8∗	T	A	G	T	G	C	T	36.83	33.51	35.10	42.62	42.02	42.44	.00343
9	T	A	G	T	G	t	T	0	0	0	0.37	0.42	0.38	.09237
10	T	A	T	T	G	C	T	0	0	0	0.92	2.1	1.28	.00207
11∗	T	A	t	T	G	t	T	39.09	40.05	39.59	24.72	23.53	24.36	<.00001
12	T	del	G	T	G	C	T	0	0	0	0.18	0	0.13	.33153
13	T	del	t	T	G	t	T	0	0	0	0.74	1.26	0.90	.01005

*Note:* The major variant of a polymorphism is marked with a capital, and the minor variant with a lowercase letter. The haplotypes are in concordance with haplotype networks (Fig. 2).

a The difference between the Danish and Estonian-Finnish (Est/Fin) sample sets is calculated by the χ^2^-test.

∗ Three core haplotypes.

**Supplemental Table 4:** Frequencies of the minor alleles of genotyped SNPs in the *CGB5* and *CGB8* genes in the subgroups of male and female partners of the couples with RM compared with fertile female controls in the Danish (n = 569) and Estonian (n = 194) and Finnish sample sets (n = 185).

Polymorphism	Estonians MAF (%)			Finns MAF (%)			Danes MAF (%)		
	Fertile controls, n = 95	RM patients, n = 99		Fertile controls, n = 100	RM patients, n = 85		Fertile controls, n = 119	RM patients, n = 450	
		Females, n = 64	Males, n = 35		Females, n = 45	Males, n = 40		Females, n = 240	Males, n = 210
c5-155	13.16	8.59	7.14	11.50	5.56	7.69	7.14	6.60	4.52
c5-142	11.50	8.59	7.14	13.00	5.56	7.69	7.14	6.60	4.52
c5+1038	14.47	8.59	10.00	14.00	5.43	10.00	7.56	8.97	5.71
c8+1045	0.53	0.78	0	3.13	0	0	0.43	0.72	0

**Supplemental Table 5:** Common variants in *CGB8* promoter and 5′ untranslated region in Danes (n = 569).

SNP	MAF (%)		*P* value
	Fertile female controls, n = 119	RM patients, n = 450	
c8-287	30.74	29.52	.85
c8-226	1.79	0.94	.34
c8-186	26.84	26.38	.83
c8+105	3.46	2.77	.83
c8+108	25.97	26.19	.94
c8+301	2.88	3.14	.83

*Note:* Association *P* values were calculated by the Cochran-Armitage test for trend.

**Table 1:** Polymorphisms identified in *CGB5* and *CGB8* in the Danish sample set in comparison with individuals from Estonia and Finland.

SNP, relative to mRNA start site^a^	Allele major/minor^b^	MAF (%) in sample set		*P* for population comparison
		Danish (n = 569)^d^	Estonian/Finnish^c^ (n = 379)^d^	
Genotyping data				
*CGB5*				
c5-155	G/C	5.94	9.92	.001
c5-142	T/A	5.94	10.58	<.001
c5+1038	C/T	7.45	11.38	.004
*CGB8*				
c8+1045	C/T	0.52	1.09	.137
Resequencing data (from −350 bp to +400)				
*CGB8*				
c8-287	T/C	29.97	25.21	.021
c8-226	A/del	1.16	0	N/A
c8-196	G/A	S(Co)	0	N/A
c8-186	G/T	26.61	39.67	<.001
c8-4	T/A	0	0.41(Pa)	N/A
c8+105	G/C	3.23	2.45	.430
c8+108	C/T	26.10	39.54	<.001
c8+135	G/A	S(Co)	0	N/A
c8+276	G/C	S(Co)	0	N/A
c8+301	T/A	3.23	5.84	.021

*Note:* N/A = not applicable.

a An SNP code includes gene name and position of the polymorphism relative to mRNA transcription start according to GenBank reference: NM_033183.2 GI:146229337 for *CGB8*.

b Alleles at the coding strand.

c Data from discovery study (19).

d The full genotyped sample comprising females and males from couples with RM and fertile female controls; allelic distribution of all investigated *CGB5* and *CGB8* polymorphisms was in HWE in the full samples as well as in the subsamples of RM patients and controls. S: singleton SNP carried by one heterozygous individual; Pa: detected only among RM patients; Co: detected only among fertile controls with no miscarriages.

**Table 2:** Frequencies of the minor alleles of c5-155 and c5-142 SNPs in the *CGB5* promoter among the women and men from couples with RM and fertile female controls in the Danish, Estonian, and Finnish sample sets.

Sample size (RM cases/fertile controls)	c5-155				c5-142			
	MAF (%)		*P* value^a^	OR (95% CI)	MAF (%)		*P* value^a^	OR (95% CI)
	Fertile controls	RM patients			Fertile controls	RM patients		
Estonians, n = 194 (99/95)	13.16	8.08	.083	0.54 (0.27–1.1)	13.16	8.08	.083	0.54 (0.27–1.1)
Finns, n = 185 (85/100)	11.50	6.55	.129	0.58 (0.29–1.19)	13.00	7.74	.131	0.52 (0.24–1.13)
Danes, n = 569 (450/119)	7.14	5.62	.369	0.77 (0.44–1.37)	7.14	5.62	.369	0.77 (0.44–1.37)
Meta-analysis across three studies (634/314)^b^			.021	0.64 (0.44–0.94)			.021	0.66 (0.45–0.94)

a Association *P* values were calculated by the Cochran-Armitage test for trend.

b Meta-analysis was performed using the inverse-variance method implemented under the fixed-effects model; estimation of the combined OR and statistical significance of association takes into account the sample sizes of each individual contributing study.
